# Nitrogen Removal in Oligotrophic Reservoir Water by a Mixed Aerobic Denitrifying Consortium: Influencing Factors and Immobilization Effects

**DOI:** 10.3390/ijerph16040583

**Published:** 2019-02-17

**Authors:** Hanyue Wang, Tong Wang, Shangye Yang, Xueqing Liu, Liqing Kou, Tinglin Huang, Gang Wen

**Affiliations:** 1 Key Laboratory of Northwest Water Resource, Environment and Ecology, MOE, Xi’an University of Architecture and Technology, Xi’an 710055, China; leaf_soinlove@163.com (H.W.); 18829029700@163.com (T.W.); 467617300@163.com (S.Y.); 18092487820@163.com (X.L.); lqkkou@163.com (L.K.); 2 Shaanxi Key Laboratory of Environmental Engineering, Xi’an University of Architecture and Technology, Xi’an 710055, China

**Keywords:** Nitrogen pollution, aerobic denitrification, oligotrophic, source water, immobilization

## Abstract

Nitrogen pollution in reservoirs has received increasing attention in recent years. Although a number of aerobic denitrifying strains have been isolated to remove nitrogen from eutrophic waters, the situation in oligotrophic water environments has not received significant attention. In this study, a mixed aerobic denitrifying consortium screened from reservoir samples was used to remove nitrogen in an oligotrophic denitrification medium and actual oligotrophic source water. The results showed that the consortium removed 75.32% of nitrate (NO_3_^−^-N) and 63.11% of the total nitrogen (TN) in oligotrophic reservoir water during a 24-h aerobic cultivation. More initial carbon source was helpful for simultaneous removal of carbon and nitrogen in the reservoir source water. NO_3_^−^-N and TN were still reduced by 60.93% and 46.56% at a lower temperature (10 °C), respectively, though the rates were reduced. Moreover, adding phosphorus promoted bacterial growth and increased TN removal efficiency by around 20%. The performance of the immobilized consortium in source water was also explored. After 6 days of immobilization, approximately 25% of TN in the source water could be removed by the carriers, and the effects could last for at least 9 cycles of reuse. These results provide a good reference for the use of aerobic denitrifiers in oligotrophic reservoirs.

## 1. Introduction

In recent years, with the limitations of the groundwater extraction, reservoirs are gradually becoming the major water supply sources in many big cities [1]. However, human activities and economic development have led to widespread nitrogen pollution in natural waters, including lakes and reservoirs. The excess nitrogen in reservoir waters not only causes eutrophication [2], but also enhances the formation of nitrogen-containing disinfection byproducts (N-DBPs) during chlorination, which poses a great risk to human health [3,4]. In 2002, the State Environmental Protection Administration of China (SEPA) promulgated stricter Environmental Quality Standards for Surface Water (GB3838-2002), in which the concentration of the total nitrogen in Grade III source water is required to be less than 1.0 mg/L. However, most of the reservoirs in China fail to meet this standard. Therefore, removing nitrogen, especially nitrate, from natural water has become an important problem that requires solutions urgently.

For in situ nitrogen removal, compared with physical or chemical methods, bioremediation is considered to be the most cost-effective and efficient [5,6,7,8]. Generally, bioremediation involves the use of denitrifying strains to remove nitrogen. Although conventional theory suggests that denitrification is a strictly anaerobic process, an increasing number of denitrifiers have been screened under aerobic conditions in recent years [9,10,11,12,13], which suggests that it should be possible to use aerobic denitrifying strains to remove nitrogen and improve water quality.

Several aerobic denitrifiers have been isolated and identified, including *Acinetobacter* sp. HA2 [14], *Pseudomonas stutzeri* PCN-1 [15], *Vibrio diabolicus* SF16 [16], *Enterobacter cloacae* HNR [17], *Pseudomonas tolaasii* Y-11 [18], *Pseudomonas stutzeri* XL-2 [19], etc. However, these strains were cultivated in nutrient-rich medium, and thus, can only serve as a reference for wastewater treatment.

Recently, several research groups have concentrated on aerobic denitrifying strains that can adapt to oligotrophic environments. For example, *Acinetobacter* sp. strain Y16 could efficiently remove ammonium at a low temperature and C/N ratio [20]; *Janthinobacterium* sp. strain M-11 was shown to remove 98%, 89%, and 89% of the ammonia, nitrate, and nitrite under cold (2 °C), low nutrient conditions [21]; and *Acinetobacter harbinensis* strain HITLi7^T^ was reported to achieve heterotrophic nitrification and aerobic denitrification at 2 °C and a low C/N ratio [22]. In our previous study, we screened and cultivated oligotrophic aerobic denitrifiers and isolated a number of denitrifiers that could remove nitrate efficiently under low nutrient conditions from reservoir source water [11,23,24,25,26,27]. But compared with pure cultured strains, the mixed cultured ones, which have seldom been investigated, may be more efficient in nitrogen removal and more adaptable to various carbon source and difficult conditions [28,29,30]. Moreover, almost all of the denitrifiers in these studies were cultivated in denitrification medium, which is quite different from actual source water.

Microbial immobilized technology has been widely used for the biological treatment of wastewater for decades [31,32,33,34], and it is believed that compared to general biological methods, immobilized microorganism treatment could be more effective and even be able to intensify the denitrification performance [35]. Some researchers have stated that the limited oxygen transfer rate inside the carriers of immobilized cells, as a deficiency of immobilized cells, could also be considered as a superiority to some degree ; the gradient concentration of dissolved oxygen in the inner spaces of the carriers would provide an effective area for accumulated nitrite reduction during aerobic denitrification [36]. However, this technology has barely been applied for improvement of oligotrophic source water, resulting in difficulties for practical engineering applications.

In this study, the denitrification characteristics of the mixed microbial consortium were studied in both oligotrophic medium and actual reservoir source water. The effects of several factors on nitrogen removal in source water were investigated, including the initial total organic carbon (TOC), low temperature, and the addition of phosphorus. In addition, the denitrification effects of the consortium immobilized in polyurethane foam cubes was studied to determine the optimal immobilized time and reusability.

## 2. Materials and Methods

### 2.1. Strains and Media

The mixed aerobic denitrifying consortium used in this study consisted of five bacterial strains isolated from the Zhoucun reservoir in Shandong Province, China (34.95° N, 117.69° E). Previous studies demonstrated that these strains, *Acinetobacter* sp. G107, *Acinetobacter* sp. 81Y, *Zoogloea* sp. N299, *Acinetobacter* sp. ZMF2, and *Novosphingobium* sp. ZHF2, have aerobic denitrification activity and can remove nitrate under oligotrophic conditions [37].

The oligotrophic denitrification liquid medium used in this study consisted of (g/L) CH_3_COONa (0.0683; equal to 20 mg/L TOC), NaNO_3_ (0.0152; equal to 2.5 mg/L nitrate, which is the approximate concentration in the source water), K_2_HPO_4_·3H_2_O (0.02), CaCl_2_ (0.01), MgCl_2_∙6H_2_O (0.01), and 2 mL of trace element solution, pH 7.0–7.5. The trace element solution contained (g/L) EDTA (0.5), ZnSO_4_ (0.1), MnCl_2_∙4H_2_O (0.05), FeSO_4_∙7H_2_O (0.1), CuSO_4_∙5H_2_O (0.1), and CoCl_2_∙6H_2_O (0.05).

The source water samples used to investigate the effects of several factors on nitrogen removal were collected from the Lijiahe reservoir in Xi’an, Shaanxi Province, China (34.00° N, 109.40° E) and pasteurized (heated at 70 °C for 30 min and then rapidly cooled at 4–5 °C) before use so as to eliminate the effects of other consortiums on changes of nitrogen. The quality of the water samples is presented in Table 1.

### 2.2. Nitrogen Removal Experiments

#### 2.2.1. Nitrogen Removal in Oligotrophic Medium

A mixed consortium (24 mL) containing the five purified strains in equal proportions was inoculated into 800 mL of prepared oligotrophic denitrification liquid medium, which was sterilized at 121 °C for 30 min, in 1000-mL shaker flasks. The flasks were sealed with sterile, breathable sealing membranes and incubated on a rotary shaker at 20 °C and 130 rpm (dissolved oxygen was maintained at 8–9 mg/L). Samples were taken every 8 h to measure the concentrations of nitrate nitrogen (NO_3_^−^-N), nitrite nitrogen (NO_2_^−^-N), ammonium nitrogen (NH_4_^+^-N), total nitrogen (TN), and total organic carbon (TOC), and every 2–8 h to measure the flow cytometric total cell concentration (FCM-TCC).

#### 2.2.2 Effects of Various Factors on Nitrogen Removal in Reservoir Source Water

The effects of a carbon source (at different concentrations), low temperature, and the addition of phosphorus on the nitrogen removal capacity of the mixed consortium in reservoir source water were investigated. Different amounts of sodium acetate were added to pasteurized reservoir source water as an extra carbon source, making the initial concentration of TOC increase 5, 10, 15 and 20 mg/L based on the original level, respectively. The adaptability of the consortium to low temperature was studied at 10 °C, and compared to the control group at 20 °C. To detect the effect of phosphorus, K_2_HPO_4_∙3H_2_O was added to pasteurized reservoir source water to make phosphorus concentration increase by 0.3 mg/L. All experiments used the same proportion of inoculum (about 3%, v/v), and were incubated at 20 °C (except for the temperature experiment) with shaking at 130 rpm. The concentrations of NO_3_^−^-N, TN, TOC and the total cell counts in the samples were examined at regular intervals.

#### 2.2.3. Performance of Immobilized Strains in Reservoir Source Water

**Figure 1:**
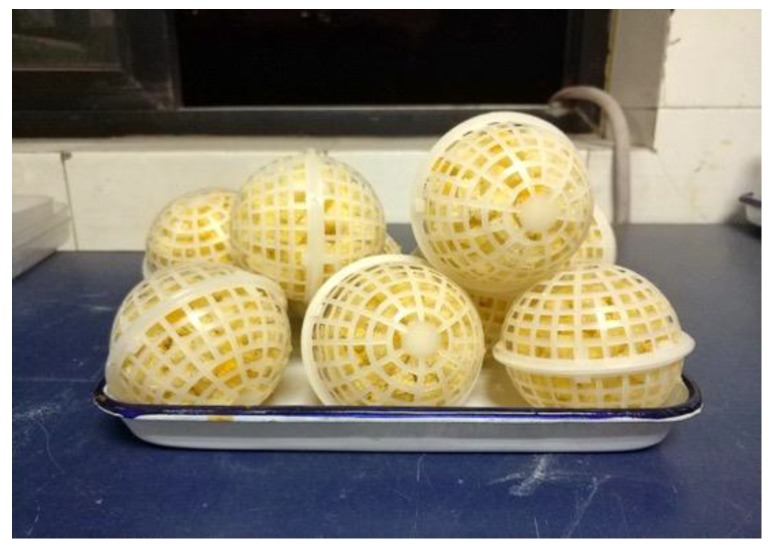
Photograph of the carriers.

Plastic balls filled with polyurethane foam cubes were used as the carriers for the consortium (Figure 1). These carriers were stored in 12 L of oligotrophic denitrification medium, and the strains were attached to the carriers by inoculation with the activated mixed consortium at regular time intervals to maintain the nitrogen removal effects, with an aerator to maintain aerobic conditions. The nutrients were replenished every 24 h. To determine the required time for immobilization, the carrier was removed from the medium after 4–9 days of cultivation, and added to 1.2 L of fresh pasteurized reservoir source water with shaking at 130 rpm. The concentrations of NO_3_^−^-N and TN in the samples were measured respectively before and after the 24-h cultivation.

In addition, polyurethane foam cubes taken before and after the 6-day cultivation and 3 cycles of reuse were analyzed by scanning electron microscopy (SEM) to observe the changes in the surface morphology of the carrier. Moreover, the carrier that was removed on day 7 was selected to verify the reusability of the immobilized strains. After performing the procedure mentioned above, the carrier loaded with the mixed consortium was transferred to another 1.2 L of the fresh reservoir source water every 24 h for several times, with shaking at 130 rpm. The initial and the final concentrations of NO_3_^−^-N and TN in the water samples were measured.

### 2.3. Analytical Methods

The total cell concentrations of the samples were determined by flow cytometric analysis. In brief, 500 μL of diluted sample was stained with 5 μL of SYBR Green I nucleic acid stain (diluted 1:100 in filtered DMSO, Invitrogen, Carlsbad, CA, USA) and incubated in the dark at 30 °C for 10 min before analysis with a BD Accuri C6 flow cytometer (BD Biosciences, San Jose, CA, USA) [38,39]. The concentrations of NH_4_^+^-N, NO_2_^−^-N, and NO_3_^−^-N were measured by using Nessler’s reagent spectrophotometry, N-(1-naphthalene)-diaminoethane spectrophotometry, and ultraviolet spectrophotometric methods, respectively. TN was determined by using alkaline potassium persulfate oxidation-UV spectrophotometry method (UV2600, UNIC, Shanghai, China). The TOC concentration was determined by using a TOC-L analyzer (Shimadzu, Kyoto, Japan). All experiments were conducted in triplicate (*n* = 3).

## 3. Results and Discussion

### 3.1. Characteristics of Nitrogen Removal in Oligotrophic Medium

**Figure 2:**
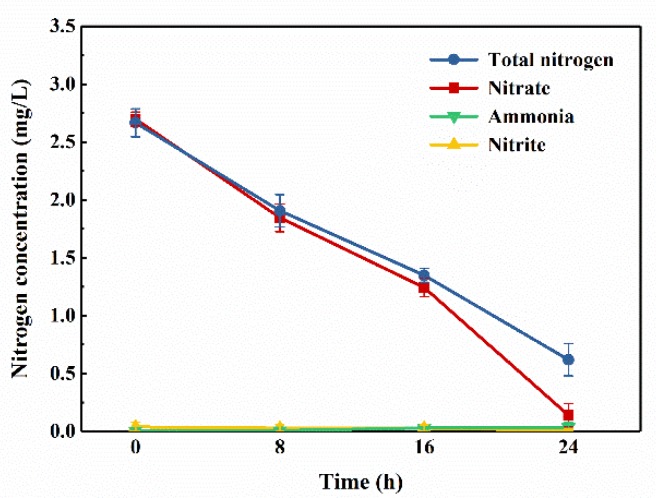
Changes in total nitrogen (TN), nitrate, ammonia, and nitrite concentrations of the mixed aerobic denitrifying consortium growth in nitrate nitrogen medium with adding 20 mg-C/L sodium acetate. Experimental conditions: T = 20 °C, 130 rpm, pH = 7.5. (The vertical lines mean ± standard deviations.)

The nitrogen removal performance of the mixed aerobic denitrifying consortium in denitrification medium containing limited nutrients are presented in Figure 2. After a 24-h cultivation, the nitrate concentration decreased from 2.70 mg/L to 0.14 mg/L, while the concentration of the TN decreased from 2.67 mg/L to 0.62 mg/L. The removal efficiencies for nitrate and TN were 94.80% and 76.78%, respectively, confirming the denitrification ability of the mixed consortium in oligotrophic medium. Furthermore, nitrite and ammonia, with their concentrations kept being < 0.1 mg/L, were barely accumulated during the cultivation.

Compared with the pure cultured strain, the mixed consortium performed better on denitrification in oligotrophic environment. According to previous studies, single strain G107, 81Y, and N299 only removed 39.90%, 33.72% and 46.79% of TN, respectively, while stain ZMF2 and ZHF2 respectively reduced around 63% and 86% of the nitrate in the denitrification medium with 30 mg-C/L, which was more effective than the results obtained in this study [11,37].

**Figure 3:**
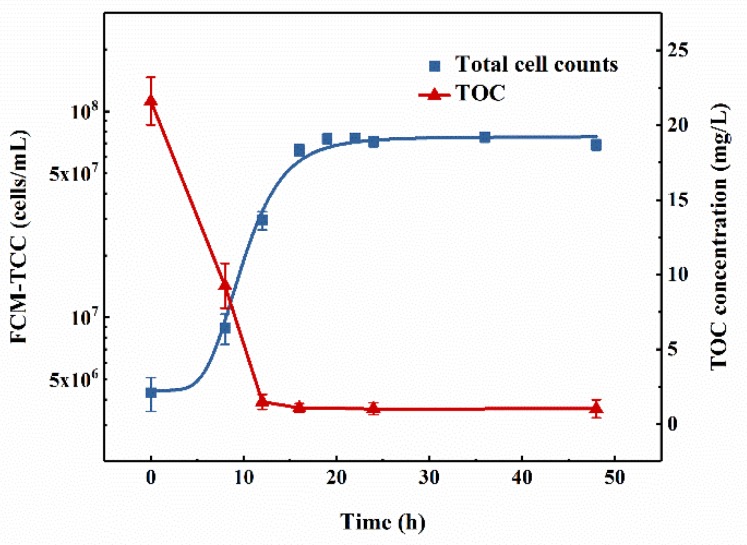
Changes in flow cytometric total cell counts (FCM-TCC) and TOC concentration of the mixed aerobic denitrifying consortium growth in nitrate nitrogen medium with adding 20 mg-C/L sodium acetate. Experimental conditions: T = 20 °C, 130 rpm, pH = 7.5. (The vertical lines mean ± standard deviations.)

The growth curve for the mixed consortium in oligotrophic denitrification medium was plotted according to flow cytometric total cell concentrations (Figure 3). The curve showed that the lag phase was about 3 h, after which an exponential phase (3–18 h) was observed, during which the total cell concentration increased rapidly from 4.0 × 10^6^ cells/mL to 7.5 × 10^7^ cells/mL. The growth rate was as high as 0.20 h^−1^. After 18 h, the density of the strains tended to remain stable, at around 7.5 × 10^7^ cells/mL.

During microbial growth, a change in the TOC concentration was observed (Figure 3**)**. At the end of the logarithmic growth phase and the beginning of the stationary growth phase, the TOC concentration decreased to 1.08 mg/L (from an initial concentration of 21.61 mg/L) and reached the minimum value at 24 h, with a removal efficiency of 95.00%. Combined with Figure 3, the time period of fastest TOC removal was observed at 0–8 h, while the time period of fastest nitrogen removal was at 8–24 h, indicating that the reduction in TOC occurred prior to log phase and the removal of nitrogen lagged slightly behind.

It was previously reported that 10 μg of a carbon source and 1 μg of a nitrogen source would provide adequate nutrients for the growth of approximately 10^8^ cells [40,41]. In this study, 800 mL of denitrification medium with an initial TOC concentration of 20 mg/L (21.61 ± 1.61 mg/L in practice) contains 17.29 ± 1.29 mg of TOC. Figure 3 showed that the final concentration of TOC was 1.08 ± 0.29 mg/L (0.86 ± 0.23 mg/800 mL). Therefore, after 24 h of cultivation, the mixed consortium removed 16.43 ± 1.52 mg of carbon source, and the total nitrogen removal could be calculated similarly. Since the total cell concentration increased to 7.5 × 10^7^ cells/mL (6.0 × 10^10^ cells in 800 mL of medium), the amount of carbon and nitrogen used for cell growth should be 6.00 mg and 0.60 mg, respectively. Based on the proposed pathways of nitrate removal by aerobic denitrifiers [13,21], except for the portions used for cell growth, 10.43 mg of carbon and 1.04 mg of nitrogen were used for denitrification. However, the nitrogen that was converted to gas could be described by TN removal in actual measurement (1.64 mg), which was higher than the theoretical value mentioned above, meaning that the mixed consortium required less nutrition to maintain cell growth and had better denitrification ability in oligotrophic nitrate nitrogen medium.

### 3.2. Nitrogen Removal in Actual Oligotrophic Reservoir Source Water

In order to examine the denitrification ability of these mixed aerobic denitrifying consortium in oligotrophic reservoir water, the effects of several factors on nitrogen removal by these strains in actual oligotrophic environment were evaluated. The mixed consortium was inoculated into pasteurized reservoir source water with different initial TOC concentrations, temperatures and phosphorus contents. The changes in the concentrations of NO_3_^−^-N, TN, total cell counts, and TOC were monitored at regular intervals.

#### 3.2.1. Effect of Carbon Source Concentration

**Figure 4:**
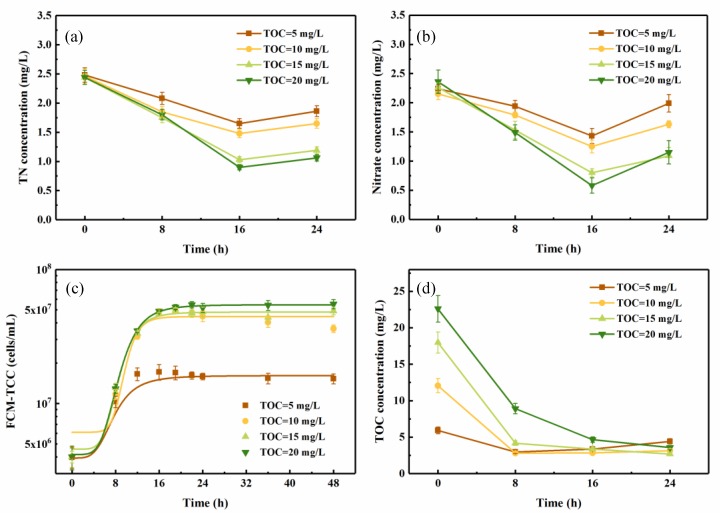
Effect of initial TOC concentration (adding 5, 10, 15, 20 mg-C/L) on the changes in (**a**) TN, (**b**) nitrate, (**c**) FCM-TCC, and (**d**) TOC concentration of the mixed aerobic denitrifying consortium growth in pasteurized reservoir source water. Experimental conditions: T = 20 °C, 130 rpm, pH = 7.5. (The vertical lines mean ± standard deviations.)

Different amounts of sodium acetate (5, 10, 15, and 20 mg-C/L) were added to the pasteurized reservoir source water as an extra carbon source. As shown in Figure 4a,b, optimal denitrification performance was achieved in source water spiked with 20 mg/L extra carbon source, and in this sample, the nitrate concentration declined from 2.36 mg/L to 0.58 mg/L in 16 h, while the TN decreased from 2.44 mg/L to 0.90 mg/L. Therefore, 75.32% of the nitrate and 63.11% of the TN were consumed during this period. In addition, the nitrogen removal capability was positively correlated with the initial concentration of TOC. After 16 h, due to the lack of nutrients, C/N ratio decreased obviously, and the cell growth gradually turned into the decline phase. Thus, with the self-decomposition of bacteria, part of the nitrogen synthesized by cell growth returned to the water samples, resulting in a slightly rise of nitrate and TN concentrations. A similar result was observed in the strain Sxf14 [42]. These data showed that in oligotrophic conditions, the mixed consortium could achieve remarkable nitrogen removal, and the effects could be improved by increasing the concentration of the carbon source, which served as not only a nutrient for cell growth, but also as an electron donor for denitrification [43,44].

The growth curves of the mixed consortium in different media (Figure 4c) showed similar trends to those observed in denitrification medium: the lag phase was about 3–4 h, followed by the exponential phase, which lasted until around 16 h, and then stationary phase. The initial total cell counts in the source water containing 5, 10, 15, and 20 mg-C/L were all at 3.0 × 10^6^ cells/mL, which increased to 1.7 × 10^7^, 4.0 × 10^7^, 4.5 × 10^7^, and 5.5 × 10^7^ cells/mL at the stable phase, respectively. Compared to the cell growth in denitrification medium with the same carbon content, lower total cell counts were observed in the reservoir water at stationary phase, as the cell growth might be limited by other necessary nutrients (calcium, magnesium, phosphorus, or other trace elements). However, the duration of log phase was somewhat shortened in the reservoir water. This shorter cell growth cycle means a faster overall proliferation rate in reservoir water, where the nutrients could be regarded as constants.

The TOC-concentration curve shown in Figure 4d was in agreement with the observed changes in cell growth, i.e., with the log phase occurring in the first 16 h of cultivation, TOC concentration declined rapidly. There was a positive correlation between the carbon removal rate and the initial TOC concentration, with 84.20%, 84.98%, 76.37%, and 43.24% of the TOC decomposed by 16 h in source water spiked with 20, 15, 10, and 5 mg-C/L, respectively. Nevertheless, the carbon source was not depleted in any test medium, as 2.5–3.5 mg/L of TOC remained in each sample, which might be due to the unassimilable organic carbon in the reservoir source water.

#### 3.2.2. Effect of Temperature

**Figure 5:**
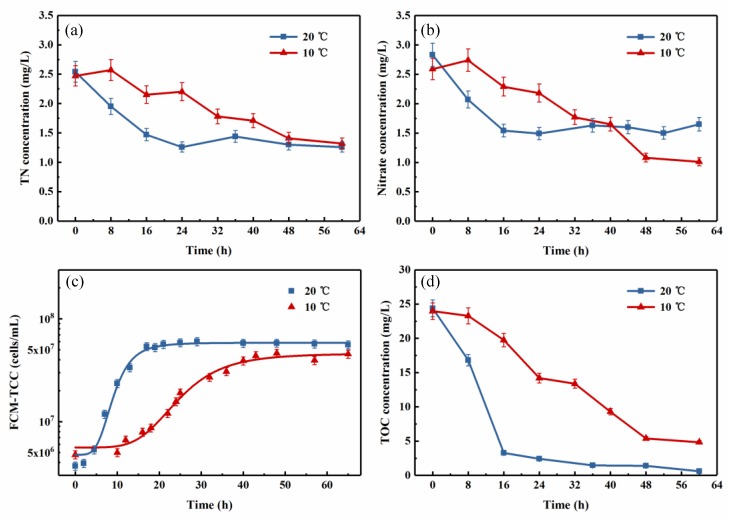
Effect of temperature (20 °C and 10 °C) on the changes in (**a**) TN, (**b**) nitrate, (**c**) FCM-TCC, and (**d**) TOC concentration of the mixed aerobic denitrifying consortium growth in pasteurized reservoir source water with adding 20 mg-C/L sodium acetate. Experimental conditions: T = 20 °C, 130 rpm, pH = 7.5. (The vertical lines mean ± standard deviations.)

To investigate the effect of low temperature on the nitrogen removal performance of the mixed consortium, two samples of pasteurized reservoir source water were inoculated with the same volume of the mixed consortium and cultivated at 20 °C and 10 °C, respectively. The changes in the concentrations of nitrogen and TOC and the total cell counts are presented in Figure 5.

Nitrogen removal performance is shown in Figure 5a,b. During the 60-h cultivation at 10 °C, the NO_3_^−^-N concentration decreased from 2.59 mg/L to 1.01 mg/L, with a removal efficiency of up to 60.93%, whereas the TN concentration was reduced by 46.56%, from 2.47 mg/L to 1.32 mg/L. Compared with the nitrogen removal at 20 °C, in which 47.52% of the NO_3_^−^-N and 50.39% of the TN were removed, there was a slightly decrease in the nitrogen removal activity at the lower temperature condition, but the final removal efficiency of TN was not obviously different, while that of NO_3_^−^-N was even higher at the lower temperature.

A similar phenomenon was observed for cell growth and TOC removal (Figure 5c,d). At 10 °C, the lag phase was prolonged to about 10 h, and the log phase was extended accordingly; thereby, the time to reach the stationary phase was increased from 16 h to about 48 h. However, although the multiplication rate was slowed, the final total cell concentration still reached 4.5 × 10^7^ cells/mL, which is nearly the same as that at “normal” temperature (20 °C). As for the TOC concentration at 10 °C, it decreased slowly, and the rate remained constantly for the first 48 h at about one third of the rate at 20 °C. Then, at the beginning of the stationary phase, it tended to be stable at about 5 mg-C/L. A lower temperature environment led to a lower removal efficiency of TOC but a comparable or higher removal efficiency of nitrogen, reflecting that low temperature could promote the simultaneous removal characteristics of carbon and nitrogen source to some degree, though the process might be longer.

Therefore, we concluded that the low temperature significantly affects the rates of cell growth and nitrogen and carbon removal, but without any obvious effects on the final denitrification, and it may be more suitable for nitrate removal.

#### 3.2.3. Effect of Phosphorus Addition

**Figure 6:**
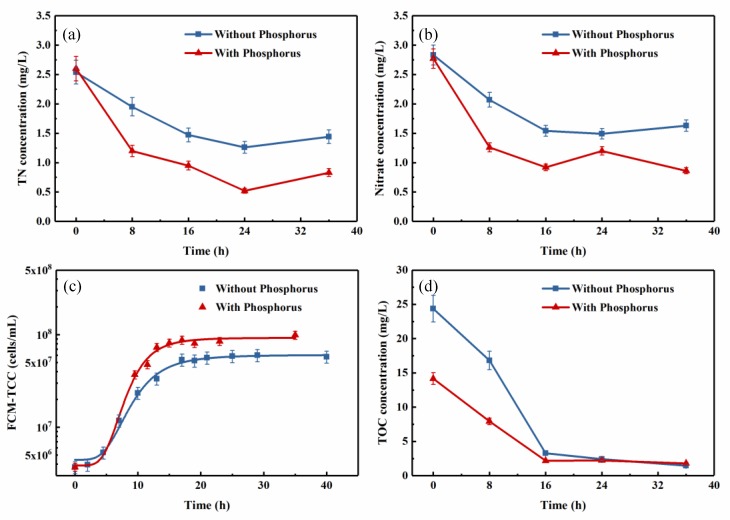
Effect of phosphorus addition (0.3 mg/L) on the changes in (**a**) TN, (**b**) nitrate, (**c**) FCM-TCC, and (**d**) TOC concentration of the mixed aerobic denitrifying consortium growth in pasteurized reservoir source water with adding 20 mg-C/L sodium acetate. Experimental conditions: T = 20 °C, 130 rpm, pH = 7.5. (The vertical lines mean ± standard deviations.)

In view of the low concentration of phosphorus in the actual reservoir source water, the effect of adding phosphorus on nitrogen removal and cell growth was examined. In the experimental samples, 0.3 mg/L of phosphorus was added to the reservoir source water, while nothing was added to the control samples; the results are shown in Figure 6.

The experimental group showed the same trends in nitrogen removal as the control group (Figure 6a,b), i.e., the nitrogen concentration declined significantly before 16 h, and then tended to be stable at 16–36 h. In the experimental group, which had less initial TOC, nitrate and TN decreased from 2.77 mg/L and 2.60 mg/L to 0.86 mg/L and 0.83 mg/L, respectively, with removal efficiencies of 68.92% and 68.08%, respectively. Compared with the control group, the nitrogen removal efficiencies were improved by approximately 20% in the experimental group, implying that the addition of phosphorus enhanced nitrogen removal by the mixed consortium.

The cell growth curves for the two groups are given in Figure 6c. The experimental group reached a stationary stage at around 14 h, which was earlier than the control group, perhaps because of the lower initial TOC concentration. However, the final total cell counts of the experimental group was nearly 1.0 × 10^8^ cells/mL, which was distinctly higher than that of the control group. This result revealed that adding phosphorus properly accelerates the cell growth and intensifies the nitrogen removal performance of the consortium.

### 3.3. Changes in the Consumed C/N Ratio

Table 2 lists the initial C/N ratios, consumed C/N ratios (ΔTOC/ΔNO_3_^−^-N and ΔTOC/ΔTN), and nitrogen removal efficiencies of all the experimental groups described in Section 3.1 and Section 3.2. It is easy to see that there is a positive correlation between the initial C/N ratios and the consumed C/N ratio. The utilization efficiency of carbon source can also be obtained from these data.

In the oligotrophic denitrification medium, the value of ΔTOC/ΔNO_3_^−^-N at 8.02 was roughly the same as the initial C/N ratio of 8.00, while in the source water, the consumed C/N ratio appeared to be much higher than the initial C/N ratio. Relatively lower utilization efficiency of carbon source in the reservoir source water might be ascribed to the oligotrophic complex multi-component conditions. Meanwhile, as the initial TOC concentration of the source water increased, the initial C/N ratio raised, and the value of ΔTOC/ΔNO_3_^−^-N increased progressively. When the extra TOC was greater than 10 mg/L, the positive correlation between these two rates increased dramatically, but the utilization efficiency of carbon source and the nitrogen removal efficiency improved significantly with more TOC contents.

As for our studies on the effects of low temperature and phosphorus addition, several conclusions may be made. Firstly, the experimental group cultured at 10 °C showed a lower ΔTOC/ΔNO_3_^−^-N and a higher initial C/N ratio than the control group did, with comparable or better denitrification effects. This verified the adaptability of the mixed consortium to low temperature conditions, perhaps because the strains we used were isolated from a reservoir with similarly low temperature water.

Secondly, to remove 1 mg of nitrate, the experimental group with phosphorus addition only consumed 6.27 mg of TOC, which was nearly one-third of that consumed in the no phosphorus control group, and even lower than that consumed in the denitrification medium. Additionally, compared with the results of the experiments described in 3.1, the experimental group added with phosphorus still had a lower ΔTOC/ΔNO_3_^−^-N ratio than the group with 10 mg/L TOC, which had almost the same initial C/N ratio as the phosphorus-added group. This result indicated that adding phosphorus could effectively raise the carbon source utilization efficiency of the mixed consortium in reservoir source water. A similar conclusion could also apply to heterotrophic denitrification in a biofilter dealing with phosphorus-limited surface water [45].

Additionally, it is worth mentioning that an aerobic denitrification strain, HITLi7^T^, has been isolated that can remove nitrate under low nutrient conditions at 2 °C with a ΔTOC/ΔNO_3_^−^-N of 4.128 [22], which is much lower than the ΔTOC/ΔNO_3_^−^-N ratio examined in this study. The reason for this might be the difference in the initial C/N ratio: strain HITLi7^T^ was cultured in medium a C/N ratio of 4, while the C/N ratio of the mixed consortium was 8. However, the denitrification effects of strain HITLi7^T^ doesn’t perform very well, and only 7.78% of the nitrate was removed. Compared to strain HITLi7^T^, the strains tested in this study could also achieve a low consumed C/N ratio (ΔTOC/ΔNO_3_^−^-N = 3.19) with a low initial C/N ratio condition (C/N = 2.64) even in source water, with 35.87% of the nitrate removed. Therefore, we can conclude that the consumed C/N ratio should not be considered without the supporting evidence of the initial C/N ratio or the removal efficiency. Specifically, these three parameters should be combined to estimate the denitrification characteristics of the target strain.

### 3.4. Nitrogen Removal Performance of Immobilized Mixed Strains in Reservoir Source Water

To maximize the denitrification performance of the immobilized mixed consortium, the optimal immobilized time and the reusability of immobilized strains were inspected. It has been reported that, among several common carriers used for immobilized bacteria, sodium alginate beads could achieve maximum TN removal efficiency while their reusability was poor. In contrast, polyurethane foam cubes and mycelial pellets performed well both in removal efficiency and reusability [36]; therefore, polyurethane foam cubes were selected as the carriers in this study.

#### 3.4.1. Determination of Immobilized Time

**Figure 7:**
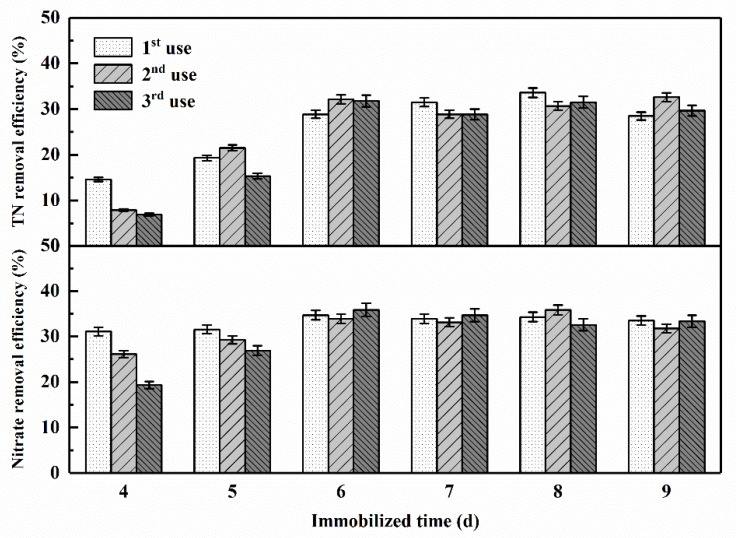
TN and nitrate removal efficiency in pasteurized reservoir source water with adding 10 mg-C/L sodium acetate by the carriers immobilized with mixed aerobic denitrifying consortium for 4–9 days. (The vertical lines mean ± standard deviations.)

Several plastic balls filled with polyurethane foam cubes were cultured in oligotrophic denitrification medium with a suspension of the activated mixed consortium. From the fourth day of cultivation, one of the carriers was taken out every 24 h, and transferred to pasteurized fresh reservoir source water with 10 mg-C/L sodium acetate addition for three times, and its denitrification performance was recorded so as to assess the optimal immobilized time. The removal efficiencies of nitrate and TN are displayed in Figure 7.

Generally, carriers cultured for 4 days showed the capacity to remove nitrogen in source water, and nearly 15% of the TN and 30% of the nitrate was degraded in the first round. However, after two cycles of reuse, there was a significantly decline in nitrogen removal, indicating that the mixed strains were not firmly attached to the carrier balls in 4-day cultivation, and thus, that the denitrification performance of the carrier is unsustainable. With the extension of the immobilized time, the nitrogen removal effects and the reusability of the carriers were obviously improved. After 6 days (or longer) of immobilization, the carriers with adhered mixed consortium tended to perform stably in terms of denitrification, and the TN removal efficiency appeared to be stabilized at around 30%, while the nitrate removal efficiency was kept at around 35%. Therefore, we concluded that at least 6 days was required for the immobilized strains to attach to this type of carrier. Similar results (4–5 days) were observed by other research teams using strain T13 in eutrophic denitrification medium [36], and the small deviations between their studies and ours might have been caused by the large differences in nutrient conditions.

**Figure 8:**
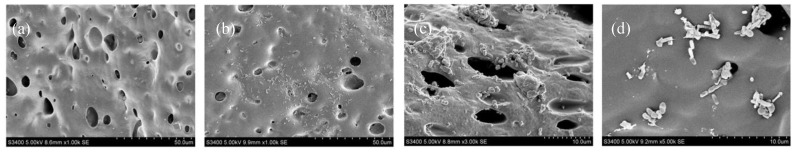
SEM micrographs of the carriers (before and after 6-day immobilization and 3 cycles of reuse): (**a**) SEM micrographs of raw carrier; (**b**) SEM micrographs of ripened carrier (×1000); (**c**) SEM micrographs of ripened carrier (×3000); (**d**) SEM micrographs of ripened carrier (×5000).

In addition, polyurethane foam cubes sampled before and after the 6-day cultivation and 3 cycles of reuse were analyzed by scanning electron microscopy (SEM). Figure 8 shows SEM micrographs of the carrier before and after immobilization.

As shown in the micrographs, the surface of the polyurethane foam cube was smooth and clean initially, while that of the “ripened” one had adhered amounts of bacteria. When viewed at higher magnification, the microgram clearly showed that most cells were adhered at the pores rather than on the surface of the polyurethane foam, which would bind the biomass more tightly to the carrier.

#### 3.4.2. The Reusability of the Immobilized Strains

To determine the reusability of a ripened carrier that was saturated with immobilized strains, the carriers cultured for 7 days were selected for the experiment, and the nitrate and TN removal efficiencies were examined during 9 cycles of reuse.

**Figure 9:**
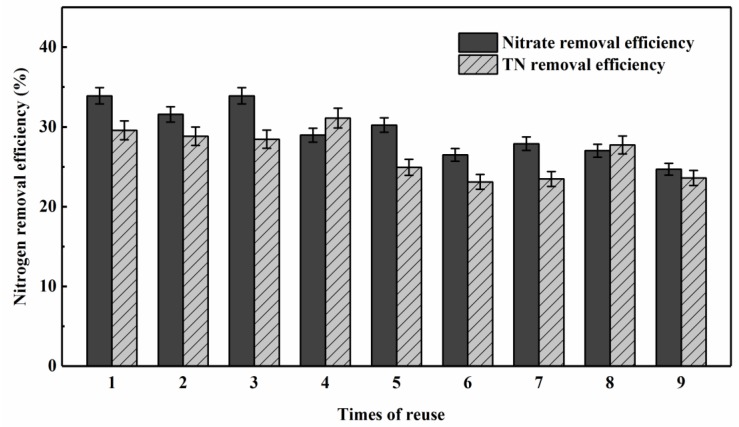
TN and nitrate removal efficiency in pasteurized reservoir source water for 1–9 times reuse by carriers immobilized with the mixed aerobic denitrifying consortium (immobilized time is 7 days). (The vertical lines mean ± standard deviations.)

As shown in Figure 9, the TN removal efficiencies were maintained at around 25%, while the removal efficiencies of nitrate were maintained at >25%, demonstrating that the ripened carriers still possessed relatively stable denitrification performance after multiple reuses. Other studies using another denitrifier immobilized on the same type of carrier showed that it could remove 40% of the TN in eutrophic denitrification medium [36], which was only 15% more than that removed in our study, with about 100 times higher in nutrients.

Compared to the free cells, the immobilized carriers could not only achieve significant and sustainable denitrification effects, but could also provide a comparatively centralized and stable microenvironment for cell attachment and proliferation, which could be beneficial for denitrifiers to be enriched and to become dominant flora in changeable actual reservoir water environment. As a preliminary study, the persistent activity of the ripened carriers demonstrated the denitrification ability of the immobilized strains in oligotrophic conditions, and provided the feasibility of using immobilized strains for the denitrification of oligotrophic reservoir waters in a real scale.

## 4. Conclusions

The main conclusions of this study are as follows:

- The mixed aerobic denitrifying consortium used in this study had remarkable nitrogen removal effects in actual oligotrophic source water, which was limited in all types of nutrients compared to the denitrification medium, and a suitable increase in the initial TOC may be beneficial for not only the denitrification effects, but also the simultaneous removal of carbon and nitrogen.
- In the reservoir source water, a lower temperature led to slower rates of cell growth and nitrogen removal; however, its influences on overall growth and nitrogen removal effects were small. Meanwhile, the addition of phosphorus facilitated cell growth, thus improving nitrogen removal performance.
- There is a positive correlation between the initial C/N ratios and the consumed C/N ratio (ΔTOC/ΔNO_3_^−^-N and ΔTOC/ΔTN) in oligotrophic environment. For this consortium, the utilization efficiency of carbon source can be encouraged by more initial carbon source, lower temperature and phosphorus addition.
- The immobilized time of the mixed consortium on carrier balls filled with polyurethane foam cubes is about 6 days, and the ripened carriers could achieve stable denitrification ability for at least 9 cycles reuse, which verified the reusability of the immobilized consortium, providing a good reference for TN removal in practical engineering applications.

## Figures and Tables

**Table 1:** Characteristics of the source water samples from Xi’an Lijiahe reservoir.

**Index**	**TN (mg/L)**	**NO_3_^−^-N (mg/L)**	**NO_2_^−^-N (mg/L)**	**NH_4_^+^-N (mg/L)**	**TP (mg/L)**	**TOC (mg/L)**	**COD_Mn_ (mg/L)**
**Value**	2.64 ± 0.23	2.59 ± 0.47	0.01 ± 0.01	0.20 ± 0.08	0.22 ± 0.06	2.45 ± 0.36	5.11 ± 0.48
**Index**	**Fe (mg/L)**	**Mn (mg/L)**	**pH**	**ORP (mV)**	**Conductivity (μS/cm)**	**Turbidity (NTU)**	**DO (mg/L)**
**Value**	0.16 ± 0.06	0.02 ± 0.02	7.60 ± 0.15	186 ± 11	200 ± 2	32.45 ± 2.74	8.49 ± 0.48

**Table 2:** Changes in carbon and nitrogen during the cultivation of the mixed aerobic denitrifying consortium growth at different media.

Experimental Conditions		Initial	Final		Removal Efficiency (%)		
		C/N (mg/mg)	ΔTOC/ΔNO_3_^−^-N (mg/mg)	ΔTOC/ΔTN (mg/mg)	NO_3_^−^-N	TN	TOC
Denitrification medium		8.00	8.02	10.03	94.76	75.83	95.00
Source water	5 mg/L TOC	2.64	3.19	3.09	35.87	33.47	43.24
	10 mg/L TOC	5.62	10.31	9.41	41.59	39.84	76.37
	15 mg/L TOC	7.96	10.50	10.85	64.44	57.79	84.98
	20 mg/L TOC	9.58	10.71	12.37	75.32	63.11	84.20
	20 °C / Without P	8.61	17.09	17.89	47.52	50.39	94.01
	10 °C / Without P	9.25	11.04	15.33	63.17	48.64	79.84
	20 °C / With P	5.11	6.27	6.77	68.92	68.08	84.60
HITLi7^T^ Denitrification medium/2 °C		5.30	4.13	-	7.78	-	5.89
